# fNIRS vs. EEG in audiological diagnostics: novel approaches to recording brain responses to auditory stimulation

**DOI:** 10.3389/fmed.2025.1646364

**Published:** 2025-12-05

**Authors:** Tomas Mimra, Martin Augustynek, Lukas Klein

**Affiliations:** 1 Department of Cybernetics and Biomedical Engineering, Faculty of Electrical Engineering and Computer Science, VSB–Technical University of Ostrava, Ostrava, Czechia; 2 ENET Centre - CEET, Faculty of Electrical Engineering and Computer Science, VSB - Technical University of Ostrava, Ostrava, Czechia

**Keywords:** fNIRS, BERA, EEG, evoked potentials, hemodynamic response, auditory stimulation, audiological diagnostics

## Abstract

**Background:**

Electroencephalography (EEG) is the traditional method for Auditory Evoked Potentials (AEPs) like Brainstem Auditory Evoked Response (BERA), offering excellent temporal resolution but facing limitations in portability and patient comfort. This study investigates functional Near-Infrared Spectroscopy (fNIRS) as a non-invasive, hemodynamic-based alternative.

**Methods:**

We performed simultaneous EEG and fNIRS measurements on healthy volunteers to compare responses to two auditory stimuli: rapid ”clicks” (*n* = 20) and slower, complex “warbles” (*n* = 34). Data were analyzed using correlation, *t*-tests, a General Linear Model (GLM), and Principal Component Analysis (PCA) to assess and compare signal quality and robustness.

**Results:**

For rapid click stimulation, EEG demonstrated its superiority in detecting millisecond-scale Jewett waves. While fNIRS showed transient features in temporal proximity to auditory brainstem responses (ABRs), it could not resolve these fast potentials. For the slower warble stimulation, fNIRS excelled, detecting significant hemodynamic changes in all participants. A comprehensive analysis, including a repeated-measures Analysis of Spatial Specificity (ANOVA), confirmed a highly structured, repeatable, and spatially specific response, arguing against systemic artifacts and supporting a neural origin.

**Conclusion:**

EEG remains the indispensable tool for rapid auditory brainstem diagnostics. However, fNIRS shows significant potential for measuring robust cortical responses to slower, more complex auditory stimuli. With advantages in comfort and portability, fNIRS can serve as a valuable complement to EEG, particularly in clinical scenarios where EEG application is constrained, such as in pediatric or mobile audiological assessments.

## 1 Introduction

Auditory Evoked Potentials (AEPs) constitute a crucial diagnostic tool for evaluating the integrity of the auditory pathway. The most frequently employed method is Brainstem Evoked Response Audiometry (BERA), which utilizes electroencephalography (EEG) to detect electrical activity in response to auditory stimuli. However, in recent years, there has been a growing interest in alternative recording methods that are less invasive, more portable, and better suited for specific patient populations. One such method is functional Near-Infrared Spectroscopy (fNIRS), which measures changes in the concentration of oxy- and deoxyhemoglobin in the brain as a response to functional activation.

This study aims to verify whether the fNIRS method is capable of capturing evoked potentials similarly to EEG and whether it can be used as a complement or alternative. Two different stimulation paradigms were tested: the first utilized traditional click stimuli with a high repetition rate, while the second employed complex acoustic sequences of the warble type. Measurements were performed in parallel using EEG and fNIRS electrodes in healthy volunteers.

The main contribution of this study lies in the systematic comparison of the two methods during a single measurement session, with the aim of comparing their ability to capture auditory evoked potentials under different stimulation regimes. The uniqueness of the approach lies not only in the parallel application of EEG and fNIRS but also in the application of advanced analytical methods such as PCA, correlation analyses, and t-tests.

The findings of this study indicate that fNIRS has the potential to be a valid alternative or complement to EEG, particularly in cases where a higher degree of comfort, device portability, or use outside standard laboratory settings is crucial.

## 2 State of the art

This section comprehensively reviews the current methodologies and applications of two pivotal neurodiagnostic tools in auditory research: Brainstem Auditory Evoked Response (BERA) and functional Near-Infrared Spectroscopy (fNIRS).

### 2.1 State of the art of BERA

BERA, or Brainstem Auditory Evoked Response, is a diagnostic test employed to assess the auditory pathway from the ear to the brainstem. It involves the placement of small electrodes on the scalp and earlobes to measure electrical activity in response to auditory stimuli delivered to the ear. The recorded electrical activity is subsequently analyzed to evaluate auditory function and diagnose hearing impairments.

One of the key principles of BERA is the manipulation of stimulus intensity and repetition rate to elicit specific components of the auditory evoked potential (AEP). According to the study by Radeloff et al. (1), different AEP components can be evoked using specific stimulus parameters, such as stimulus intensity and repetition rate. For instance, the peak latency of wave V of the AEP component can be utilized to assess the integrity of the auditory nerve, while the interpeak latency between waves I and III can be used to evaluate the integrity of the auditory brainstem.

Another principle underlying BERA is the use of electrode placement to localize the source of the AEP. According to the study by Jerger et al. (2), electrode placement can be used to identify the origin of the AEP along the auditory pathway, from the cochlea to the brainstem. By analyzing the latency and amplitude of the AEP at individual electrode sites, the site of a potential hearing disorder can be determined.

According to the study by Caetes et al. (3), BERA is a useful diagnostic tool for various hearing disorders, including hearing loss, auditory neuropathy, and auditory processing disorder. The authors state that BERA can provide objective measures of auditory function, which can be beneficial in the diagnosis and monitoring of these conditions.

Furthermore, a study by Gelanay et al. (4) found that BERA is effective in identifying auditory processing deficits in children with Attention-Deficit/Hyperactivity Disorder (ADHD). The authors suggest that BERA may be a useful tool for identifying auditory processing deficits in other clinical populations as well.

Overall, BERA stands as a valuable diagnostic tool for assessing auditory function and identifying a range of hearing disorders.

### 2.2 State of the art of fNIRS

Understanding speech perception at the cortical level is a crucial area of research, particularly for individuals with hearing impairments. Functional near-infrared spectroscopy (fNIRS) has emerged as a valuable neuroimaging tool due to its non-invasive nature, compatibility with hearing devices, and resistance to motion artifacts. Recent studies have employed fNIRS to investigate neural responses to speech processing in different populations, including adults and children. This section provides an overview of three significant studies utilizing fNIRS to examine cortical activity associated with speech perception.

A study by Blint et al. (5) explored cortical responses to changes in voice pitch using a combination of fNIRS and electroencephalography (EEG). In this study, 20 normal-hearing participants were exposed to sequences of vowels with varying prosodic contours. The integration of fNIRS and EEG allowed for the simultaneous measurement of hemodynamic and electrophysiological responses.

Results revealed that pitch changes elicited right-lateralized activity in the superior temporal cortex. EEG data identified two distinct adaptation mechanisms: an early bilateral sensory adaptation and a later right-hemispheric attention-related adaptation. The study demonstrated the effectiveness of combining fNIRS and EEG to map cortical responses to prosodic variations, highlighting the right hemisphere's critical role in speech melody perception. These findings provide a foundation for improving auditory processing in cochlear implant users.

Lawrence et al. (6) investigated cortical responses to speech intelligibility in normally hearing children aged 6–13 years. The study employed fNIRS to measure activity in the superior temporal and inferior frontal cortices while participants listened to sentences with varying levels of intelligibility. The goal was to determine whether neural responses could serve as objective markers of speech comprehension.

Findings indicated a dominant left-hemisphere activation in the superior temporal cortex, which increased with higher speech intelligibility. Additionally, the study observed deactivation in right posterior temporal regions under more challenging listening conditions. These results suggest that fNIRS can effectively assess speech processing in children, particularly in cases where behavioral assessments may be unreliable. The study's implications extend to the evaluation of language development delays and the assessment of hearing device efficacy in pediatric populations.

Steinmetzger et al. (7) examined cortical activation during speech comprehension tasks in normal-hearing adults. The study utilized an adapted clinical speech test, the Oldenburg Sentence Test (OLSA), and fNIRS to measure responses in the temporal, occipital, and prefrontal cortices. Speech stimuli were presented in four conditions: speech-in-quiet, speech-in-noise, audiovisual speech, and visual speech (lipreading).

The results revealed bilateral temporal activation for auditory conditions, while visual speech primarily engaged the occipital cortex. The audiovisual condition activated both temporal and occipital regions, reinforcing the integration of auditory and visual information in speech perception. Notably, prefrontal cortex activation increased under noisy conditions, indicating higher listening effort. This study confirmed the applicability of clinically inspired speech tasks in fNIRS research and provided insights into cognitive load during speech comprehension.

These studies underscore the versatility of fNIRS in speech perception research. The findings highlight the role of the superior temporal cortex in processing pitch changes, speech intelligibility, and multimodal speech integration. Furthermore, fNIRS proves to be a promising tool for evaluating auditory processing in populations where traditional behavioral assessments are challenging, such as young children and cochlear implant users. Future research should focus on extending these methodologies to clinical applications, refining assessment protocols, and exploring the neural mechanisms underlying speech perception in diverse hearing conditions.

## 3 Methodology

This section outlines the comprehensive experimental design and analytical procedures employed in this research. This study aims to compare the capabilities of EEG and fNIRS in recording auditory nerve responses to acoustic stimuli. We build upon the knowledge summarized in Section 2. Evoked potentials are recorded in parallel using EEG and fNIRS from a participant stimulated with acoustic signals. The data are subsequently processed with appropriate filters and computations. The work is divided into two separate studies.

### 3.1 Participant selection

This subsection details the criteria for the recruitment and selection of participants for the experiment. For this study, fifty-four participants (30 males, 24 females) were recruited via university announcements and word-of-mouth. The inclusion criteria stipulated an individual with no history of audiological or neurological pathologies. The participant's age was between 20 and 40 years, an age range selected to minimize the potential confounding effects of presbycusis and tinnitus, which are more prevalent in older individuals (8). Although formal audiometric testing was not conducted, participants reported normal hearing and no tinnitus, thus, major confounding effects due to presbycusis or hearing loss are unlikely. The participant was fully informed of all potential study-related risks and provided written infor med consent prior to participation. Potential risks, though minimal, included the possibility of temporary hearing threshold shift, mild nausea, or mild vertigo. No participants were excluded after enrollment all datasets were included in the final analysis.

### 3.2 Hardware and setup

The following subsections describe the instrumentation and configuration used for data acquisition and stimulus delivery.

#### 3.2.1 Biosignal acquisition system

The core of our recording setup was the biosignal amplifier, chosen for its multimodal capabilities. The g.Nautilus system served as the biosignal amplifier for this research, capable of interfacing with both EEG electrodes and the fNIRS sensors. (9, 10)

For EEG acquisition, the g.Nautilus offers software-selectable sensitivity levels of ±2.25 V, ±1.125 V, ±750 mV, ±562.5 mV, ±375 mV, and ±187.5 mV. Communication is established via a wireless 2.4 GHz ISM band interface, and the device includes 8 digital trigger inputs. Power is supplied by an internal lithium-ion battery, providing an operating time exceeding 6 h with 64 channels (and over 10 h with 8, 16, or 32 channels), supporting inductive charging according to the QI standard of the Wireless Power Consortium. The amplifier is a DC-coupled design with a noise level below 0.6μV RMS (between 1 and 30 Hz at the highest input sensitivity). It can accommodate up to 64 monopolar or 32 bipolar input channels (software-selectable with common GND and REF), with an input impedance greater than 100 MΩ (DC). The g.Nautilus is classified as Safety Class II and provides sampling rates of 250 Hz or 500 Hz, with the recommendation that only one device operate at 500 Hz within a single room (9).

For fNIRS measurements conducted with the g.Nautilus system, the integrated fNIRS module is also powered by the device's internal lithium-ion battery, offering a comparable operating duration (9). The fNIRS component provides a raw data sampling rate of 10 kHz and incorporates 8 emitters and 2 detectors (10).

#### 3.2.2 Sensors

**Figure 1:**
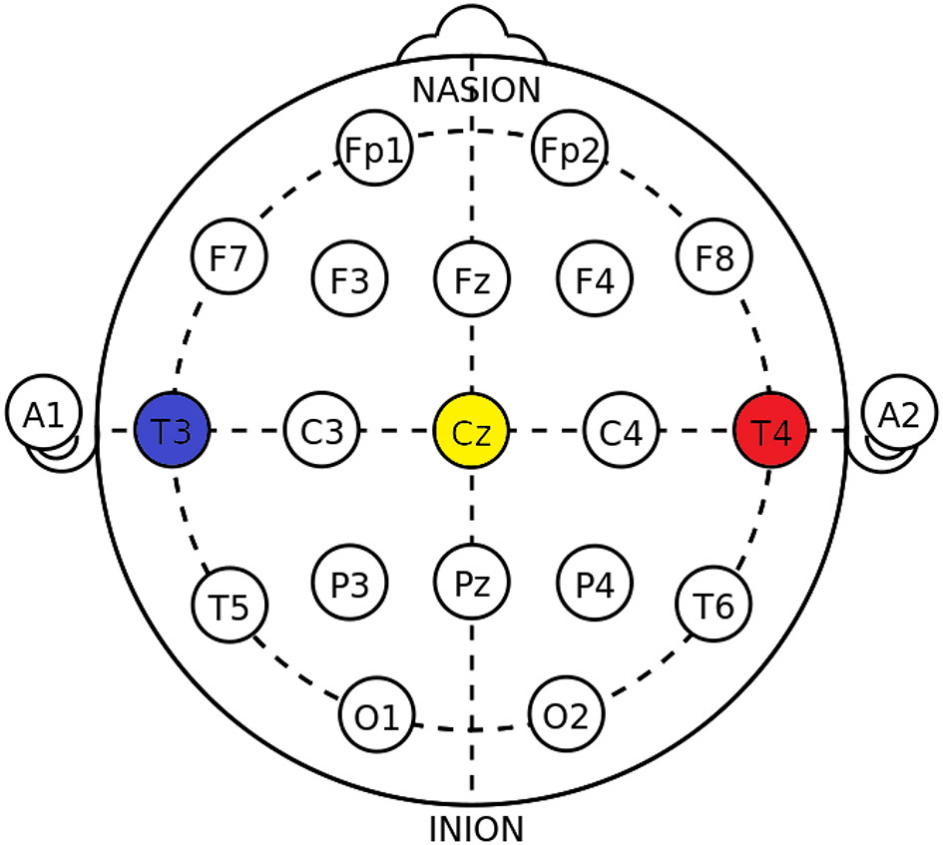
EEG electrodes Adapted from Wikipedia Contributors (12).

**Figure 2:**
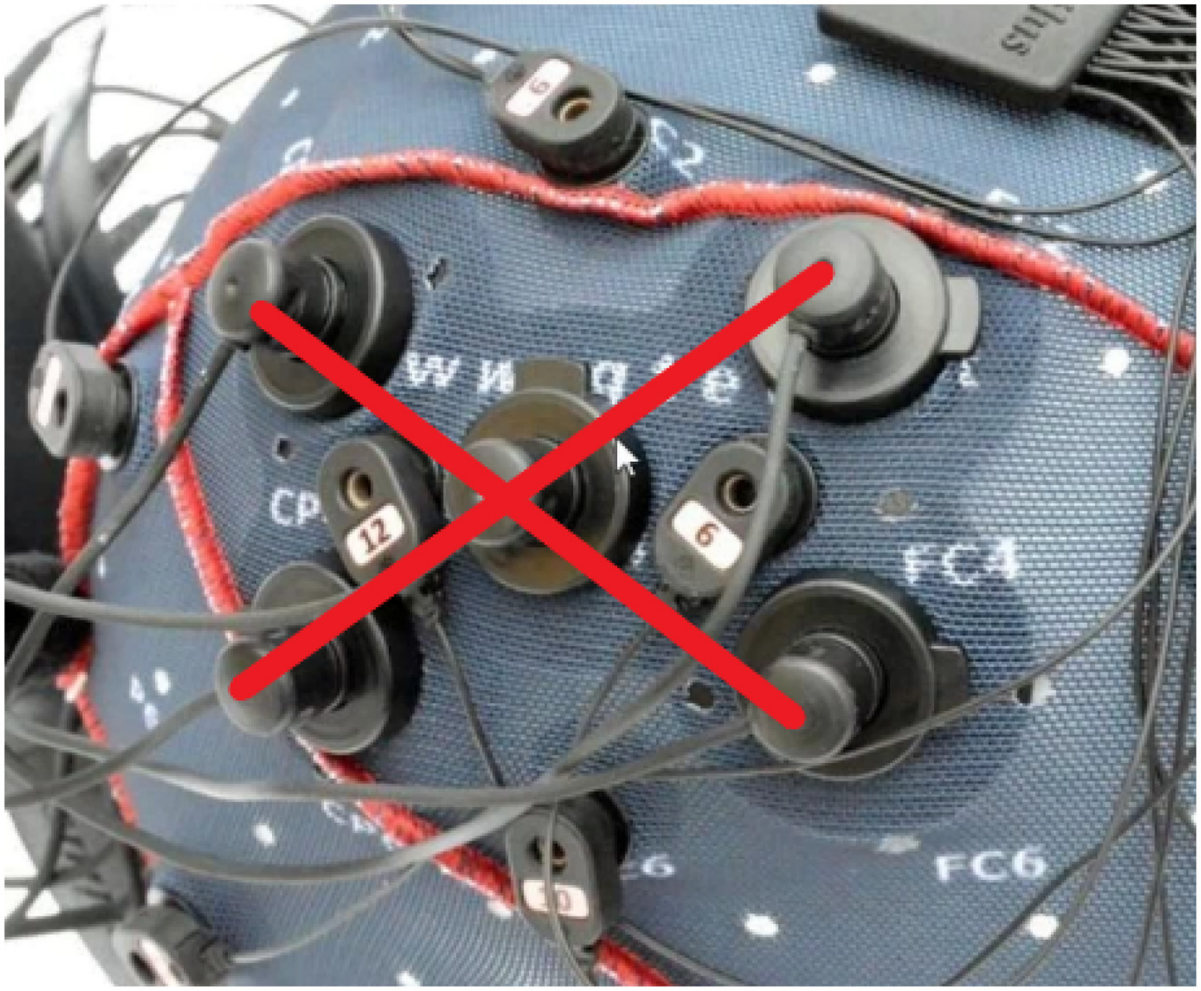
fNIRS probe placement.

Specific sensors were selected to interface with the acquisition system for optimal signal quality. The following sensor types were used in this study:

- **EEG Cup electrodes:** Reusable gel-filled electrodes that ensure high-quality signal acquisition. In our measurement setup, electrodes were positioned over the temporal lobe regions, as highlighted in Figure 1 (11).
- **fNIRS sensors:** These comprise a combination of LED emitters and photodiodes. In our study, we employed sensors arranged in an X-shaped (Figure 2 highlighted in red) configuration on the temporoparietal junction of both hemispheres. The distance between the source and detector probes was 30 mm.

Considering the trade-off between patient comfort and signal quality, cup electrodes and fNIRS sensors were selected for the present study, as it has been demonstrated by Lefler et al. (13) that cup electrodes yield more reliable data compared to disposable adhesive electrodes. Another study by Xia et al. (14) confirmed the higher accuracy of needle electrodes, albeit at the expense of reduced patient comfort.

#### 3.2.3 Auditory stimulation setup

Precise delivery and calibration of auditory stimuli were critical for this experiment. Stimulation was delivered using Sennheiser HD 280 Pro headphones. These headphones feature a nominal impedance of 64 Ω, a frequency response ranging from 8 Hz to 25,000 Hz, and a sound pressure level (SPL) of 102 dB (1 Vrms). Their sensitivity is rated at 113 dB/mW, with a total harmonic distortion (THD) of less than 0.1% (15).

The headphones were calibrated using an artificial ear (16) according to the IEC 60318 standard. Calibration showed that an input voltage of 370 mV corresponded to a click intensity of 100 dB SPL (Sound Pressure Level). Based on this reference value, the required voltages for the target stimulus intensities were computed using the following formula:


$$\begin{array}{l} dB_{1}-\text{d}{\text{B}}_{2}=20\cdot \log_{10}\left(\frac{V_{1}}{V_{2}}\right) \end{array}$$ (1)

where dB_1_ and *V*_1_ are the known reference values (100 dB SPL and 370 mV), and dB_2_ and *V*_2_ represent the target intensity and its corresponding voltage. The resulting voltages were 31.62 mV for 50 dB SPL, 79.06 mV for 70 dB SPL, and 316.23 mV for 90 dB SPL.

### 3.3 Experimental procedures

This research was structured into two distinct experimental studies, each with a unique paradigm.

#### 3.3.1 Study 1: EEG/fNIRS click paradigm

The first study utilized a classic auditory brainstem response paradigm with click stimuli. This study is designed to be as short as possible, an approach motivated by the specific needs of the pediatric population.

##### 3.3.1.1 Stimulus and procedure

The generation and presentation of stimuli followed a carefully controlled protocol. Stimulus signals were generated using Matlab (17). The stimulus was a broadband ”CLICK” implemented as a rectangular pulse with a duration of approximately 0.1 ms (18, 19). This type of stimulus activates a wide region of the cochlea, with a maximal response typically observed around 2 kHz. The stimulus repetition rate was set to 81.9 Hz.

The measurement protocol consisted of three consecutive epochs, each corresponding to one of the specified stimulus intensities (50, 70, and 90 dB SPL), delivered via the calibrated headphones. Each epoch included 1,000 CLICK stimuli, resulting in a total of 3,000 clicks per complete trial.

##### 3.3.1.2 Data processing

Post-acquisition, the raw data underwent a multi-step processing pipeline. Data processing was performed in MATLAB. Initially, the raw signal was filtered to remove noise. A band-pass filter was applied with a high-pass cutoff at 100 Hz and a low-pass cutoff at 3,000 Hz. Subsequently, the post-stimulus signal was segmented into 12 ms epochs (18). This procedure yielded 3000 epochs in total (1,000 epochs per stimulus intensity). These epochs were then categorized into three groups based on the stimulus intensity (1,000 epochs per intensity).

The data was further segregated by electrode channel for detailed analysis. This resulted in 114 distinct datasets (38 electrode channels × 3 stimulus intensities; all 38 available active electrodes were utilized for analysis), with each dataset comprising 1,000 epochs. Within each of these 114 electrode-intensity specific datasets, the 1,000 epochs were averaged. The primary output of the EEG analysis was averaged waveforms for each electrode, illustrating the response to each of the three stimulus intensities, typically presented graphically.

##### 3.3.1.3 Evaluation of waveforms

A systematic approach was used to analyze and compare the resulting waveforms from both modalities. For both EEG and fNIRS recordings, waveform evaluation focused on the detection and characterization of auditory brainstem response (ABR) components, with a particular emphasis on wave V (20). EEG data were segmented into epochs corresponding to three stimulation levels (90, 70, and 50 dB SPL). Each averaged waveform was baseline-corrected using a 0.5 s pre-stimulus interval, normalized to its peak amplitude, and analyzed for the presence and latency of waves I, III, and V. Peak detection was performed within predefined latency windows (wave I: 1.6–3.5 ms, wave III: 3.6–5.5 ms, wave V: 5.6–8.0 ms) using a minimum peak height of 0.08 and a minimum peak distance of 2 ms. Inter-peak intervals (I–III, III–V, and I–V) were also computed where applicable.

For fNIRS, the Δ[HbT] (total hemoglobin concentration change) signal was averaged over the same epochs and stimulus conditions. Baseline correction was applied using the first 2 s of the pre-stimulus period. Peak responses were identified in the same latency windows as for EEG to enable direct comparison, acknowledging the temporal resolution limits of fNIRS. Signal-to-noise ratio (SNR) was calculated for both modalities as the ratio of peak amplitude (relative to baseline mean) to the standard deviation of the baseline period.

To assess potential temporal correspondence between EEG and fNIRS responses, time-locked cross-correlation analysis was performed on the normalized waveforms, with the maximum correlation coefficient and corresponding lag extracted within a ±10 ms window. This provided an objective metric to supplement visual inspection of waveform similarity.

#### 3.3.2 Study 2: fNIRS “warble” sound paradigm

The second study introduced a novel auditory stimulus to explore hemodynamic responses with fNIRS.

##### 3.3.2.1 Stimulus and procedure

A custom auditory stimulus was designed specifically for this phase of the research, this stimulus was based on the well-known warble tone, however, its parameters were modified (21). Stimulus signals for this paradigm were generated using Matlab (17), where a custom ”warble” sound was programmed. This sound consisted of a rapid sequence of clicks designed to induce bi-directional oscillation of the headphone diaphragm. The total duration of this ”warble” stimulus was 2 s. Stimulation was delivered via the calibrated Sennheiser HD 280 Pro headphones. Stimulus intensities (50, 70, and 90 dB SPL) were set using the same methodology and formula (Equation 1) described previously.

The measurement protocol employed a block design sequence with a total duration of 92 s, structured as follows:

- 30 s of silence (baseline resting period)
- 2 s of ”warble” sound at 50 dB SPL
- 13 s of silence
- 2 s of ”warble” sound at 70 dB SPL
- 13 s of silence
- 2 s of ”warble” sound at 90 dB SPL
- 30 s of silence (post-stimulus resting period)

##### 3.3.2.2 Data processing

The fNIRS data processing was tailored to extract hemodynamic signals from the complex recordings. Processing and visualization of the functional near-infrared spectroscopy (fNIRS) data, acquired from sensor placements over regions corresponding to bilateral auditory areas, was performed in the Matlab environment (18).

The initial step involved loading the data from a .mat file. Key data, typically stored within a structure, was accessed. From this structure, changes in the concentration of oxygenated hemoglobin [Δ[HbO]] and deoxygenated hemoglobin [Δ[HbR]] for each channel were extracted. These chromophore concentration changes formed the basis for calculating changes in total hemoglobin [Δ[HbT]] according to the relationship:


$$\begin{array}{l} \Delta \left[\text{HbT}\right]=\Delta \left[\text{HbO}\right]+\Delta \left[\text{HbR}\right] \end{array}$$ (2)

**Figure 3:**
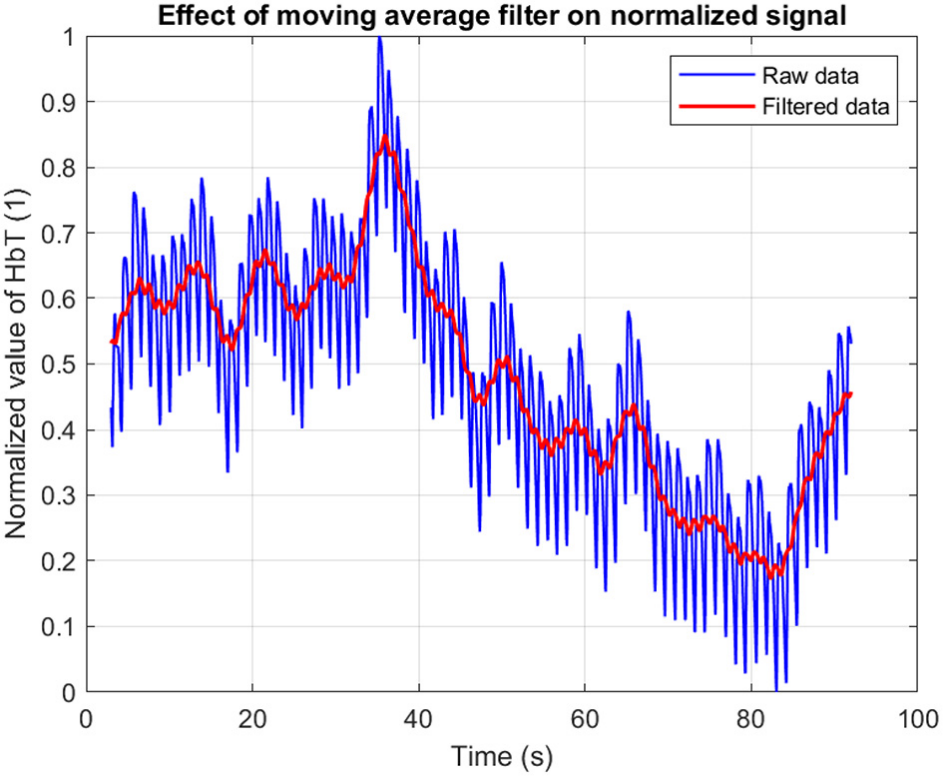
Illustration of fNIRS signal before and after moving average filtering.

The raw signal was first epoched around the stimulus onsets. To suppress high-frequency noise while preserving the underlying hemodynamic shape, a moving average filter was applied with a window size corresponding to 200 ms (Figure 3).

##### 3.3.2.3 Data evaluation

To robustly test for the presence of a stimulus-evoked response, a multi-faceted analytical strategy, or “battery of tests,” was employed on the raw fNIRS light intensity data (22). This approach aimed to build a convergence of evidence from conceptually distinct methods.

1. **Visual Inspection of Averaged Epochs:** Signals from all participants and trials were averaged to visually identify any consistent, time-locked response pattern. This block-averaging approach is a common first step for assessing response morphology (22, 23).
2. **Principal Component Analysis (PCA):** PCA was used as a data-driven method to identify the most dominant, recurring temporal patterns (components) in the epoched data (23). A consistent response should emerge as the first principal component (PC1), a technique often used for feature extraction and noise reduction (24).
3. **Statistical Modeling (GLM and Cross-Correlation):** To statistically verify the temporal relationship between the stimulus and the signal, a General Linear Model (GLM) was fitted to the data with a regressor modeling the task timing. The GLM is a standard and robust method for estimating evoked hemodynamic brain activity from fNIRS signals (22, 25). Additionally, time-locked cross-correlation was used to confirm the presence of a consistent response shape at a specific time lag relative to the stimulus.
4. **Analysis of Spatial Specificity (ANOVA):** A crucial step to distinguish a localized neural response from a global, systemic artifact (e.g., changes in blood pressure or heart rate) was a repeated-measures Analysis of Variance (ANOVA) (26). This test was performed on the response amplitudes (beta-coefficients from the GLM) with “channel” as the within-subject factor. A significant result would indicate that the response strength differs across the scalp, supporting a localized neural origin (27). *Post-hoc* pairwise t-tests were used to identify which specific channels differed.

This comprehensive strategy was designed not only to determine if a response existed but also to characterize its shape, consistency, and spatial origin, thereby validating its likely neural basis (26).

To quantitatively compare the differences in the hemodynamic response (Δ[HbT] signal) between auditory stimulation periods and silent resting periods, a correlational analysis and *t*-tests were performed. Such statistical methods are standard for investigating significant differences between conditions and relating signal features to behavioral or clinical scores (28).

### 3.4 Conditions for accurate evoked potential measurement

Ensuring the fidelity of evoked potential recordings requires strict adherence to a set of preparatory and environmental conditions. Several factors are critical for obtaining reliable recordings. Brainstem Evoked Response Audiometry (BERA) can be performed on individuals of all ages; however, specific test conditions may need to be adapted based on age. (29–31) For instance, infants and young children may require sedation or to be tested during natural sleep to minimize movement artifacts (32).

The ear canal must be clear of any obstructions (e.g., cerumen impaction) or infections that could impede sound transmission to the inner ear. Conditions such as middle ear infections or a perforated tympanic membrane can significantly affect BERA results. The participant's head position should be stabilized and maintained consistently throughout the test to ensure stable electrode placement. Participants are typically instructed to lie down or sit comfortably. The testing environment must be free from electromagnetic interference, such as that emitted by electrical devices or fluorescent lighting, which can contaminate the recordings. Furthermore, the room should be sound-attenuated to minimize interference from external noise (29).

Participants who use hearing aids should remove them before the test, as these devices can alter sound transmission and consequently affect the results. Stimulus parameters, including sound intensity, frequency, and duration, must be meticulously controlled and kept consistent throughout the testing session to ensure the accuracy of the findings (30).

Recording parameters, such as filter settings, amplification, and the number of epochs averaged, should be appropriately configured based on the participant's age and hearing status. Electrode impedance must be low (typically < 5 kΩ) and balanced across electrodes to minimize noise and artifacts in the recorded signals. Impedance levels should be checked before initiating the test and monitored if necessary. The equipment used for BERA testing must be regularly calibrated according to the manufacturer's guidelines to ensure accurate and reliable outcomes (31). Adherence to these conditions is paramount for ensuring the validity and reliability of the recordings and their subsequent diagnostic interpretation.

## 4 Results

Measurements were conducted in a designated university “smart apartment” facility, ensuring a quiet, dimly lit, and comfortable environment.

### 4.1 Study 1: EEG/fNIRS click paradigm

Data were acquired from 20 healthy volunteers. From the EEG data, a total of 2,280 averaged Auditory Brainstem Response (ABR) waveforms (114 per participant) were generated for evaluation. The primary focus of the ABR analysis was the robust identification of Wave V and the precise measurement of its absolute latency. Additionally, the presence and absolute latencies of Waves I and III were systematically assessed.

The analysis focused on identifying and characterizing Jewett waves from the EEG recordings and exploring concurrent fNIRS signal patterns. Table 1 summarizes key findings from five participants selected *post-hoc* as they exhibited the most visually distinct ABR waveforms. The results from these five selected participants demonstrate that the EEG methodology successfully recorded the expected Jewett waves, albeit with consistently increased latencies compared to standard normative values. For example, Wave V latencies were increased by up to 1.6 ms.

**Table 1:** Summary of EEG ABR and fNIRS findings in five participants.

**ID**	**Wave I presence (intensity)**	**Wave I latency deviation (ms)**	**Wave III presence**	**Wave III latency deviation (ms)**	**Wave V presence**	**Wave V latency deviation (ms)**	**Agreement between EEG and fNIRS measurement**
1	YES (EEG 70 dB)	+1.0	YES	+0.2	YES	+1.2	YES
2	YES (EEG 70 dB)	+1.0	YES	+0.7	YES	+1.3	YES
3	YES (EEG 70 dB)	+1.0	YES	+0.4	YES	+1.3	YES
4	YES (EEG 70 dB)	+1.0	YES	+0.6	YES	+1.4	YES
5	YES (EEG 70 dB)	+1.0	YES (only 70 dB)	+0.5	YES	+1.6	YES

In the EEG data from these selected cases:

- Wave I was primarily identifiable at the 70 dB SPL stimulus intensity, with its latency increased by approximately 1.0 ms.
- Wave III was generally more reliably identified than Wave I, though also exhibiting increased latencies.
- Wave V, critical for clinical assessment, was clearly identifiable. However, its latency was consistently prolonged.

**Figure 4:**
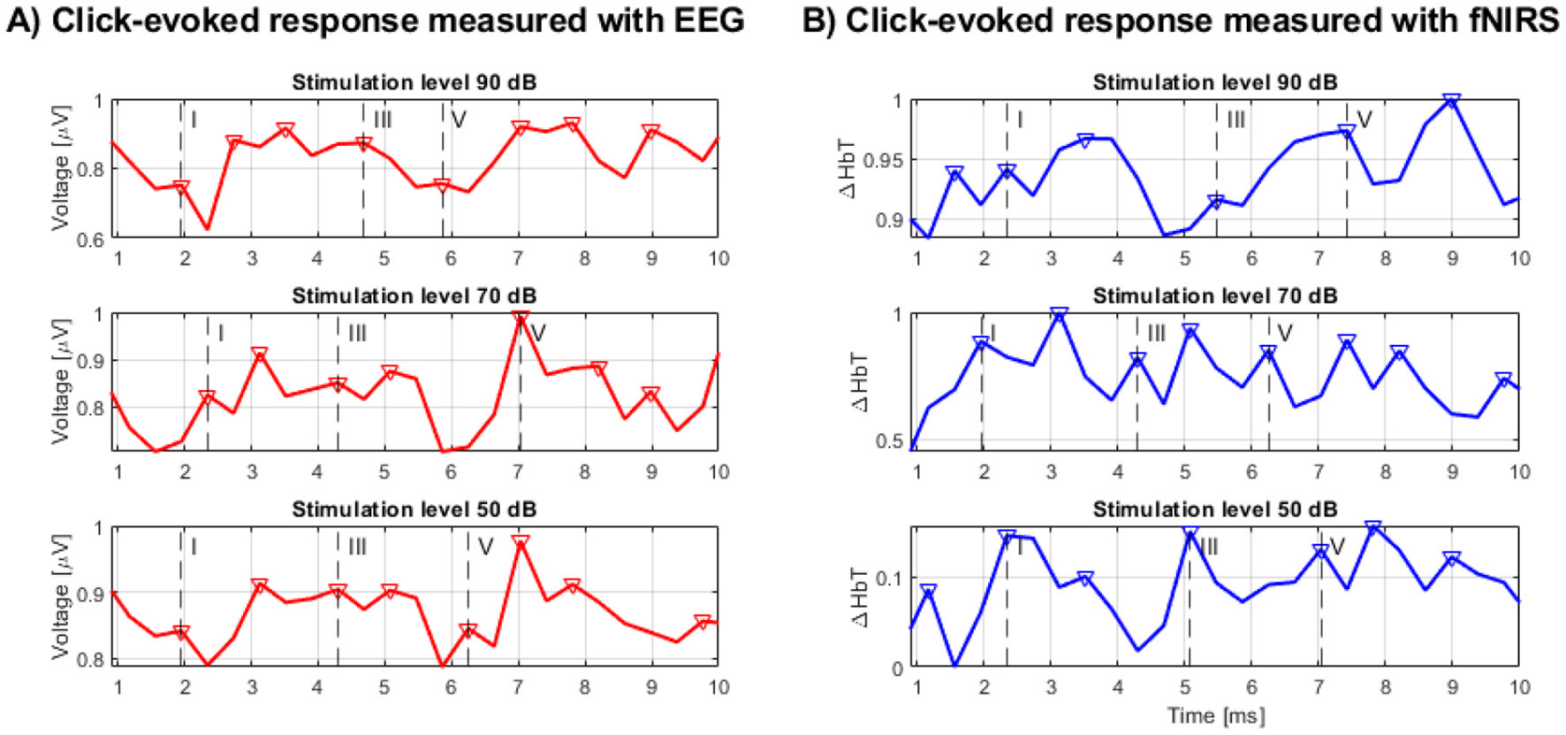
**(A)** BERA measured by EEG, **(B)** BERA measured by fNIRS.

In an exploratory analysis of the concurrent fNIRS data for these five selected participants, we visually inspected the fNIRS time courses. For the instances where Wave V was clearly present in the EEG, a transient feature in the fNIRS signal [Δ[HbT]] was observed in close temporal proximity, as shown in Table 1. This observation was made in approximately 70% of these selected participant recordings. Visual inspection of the recordings suggests that waveforms from both EEG and fNIRS methodologies were discernible (with EEG-specific ABRs shown in Figure 4A) and the concurrent fNIRS signal traces in Figure 4B), where the vertical dashed lines labeled I, III, and V indicate the detected waves I, III, and V within the specified range. Additional peaks marked with a triangle represent other detected waves with the potential to be additional responses to the stimulus.

Further quantitative analysis showed that EEG recordings demonstrated clear wave V responses at all stimulation levels, with mean SNRs of 4.97, 2.00, and 0.43 for 90, 70, and 50 dB, respectively. As expected, both amplitude and SNR decreased with stimulus intensity. In contrast, fNIRS Δ[HbT] responses showed more consistent SNR values across intensity levels—2.45 at 90 dB, 2.53 at 70 dB, and 2.20 at 50 dB—suggesting reduced sensitivity to level-dependent amplitude changes in the click paradigm.

Time-locked cross-correlation analysis revealed a moderate positive correlation between EEG and fNIRS at the highest intensity (peak *r* = 0.296, lag = +3.91 ms), negligible correlation at 70 dB (*r* = 0.009, lag = −3.91 ms), and a negative correlation at 50 dB (*r* = −0.391, lag = +3.91 ms). These results indicate that any temporal correspondence between the modalities is limited and most evident at the highest intensity, in line with the possibility of partial overlap in neural generators but also reflecting differences in the physiological origins and temporal dynamics of the signals.

Overall, the in-depth analysis concluded that EEG outperformed fNIRS in detecting fast ABR components. fNIRS responses exhibited more stable SNR across intensities but lacked consistent time-locked correspondence with EEG and are not well-suited for capturing physiological correlates of such rapidly changing neuroelectric events. These findings support the complementary use of fNIRS for assessing slower hemodynamic components in auditory processing, rather than as a direct substitute for EEG in detecting rapid auditory brainstem responses. The inherent temporal resolution of fNIRS and the nature of the hemodynamic response appear to be limiting factors. Consequently, a different approach was deemed necessary, leading to the development of Study 2.

### 4.2 Study 2: fNIRS “warble” sound paradigm

Data for the “warble” sound paradigm were acquired from 34 healthy volunteers. The comprehensive analysis of the raw fNIRS data revealed a robust, consistent, and statistically highly significant response to the auditory stimulus.

**Figure 5:**
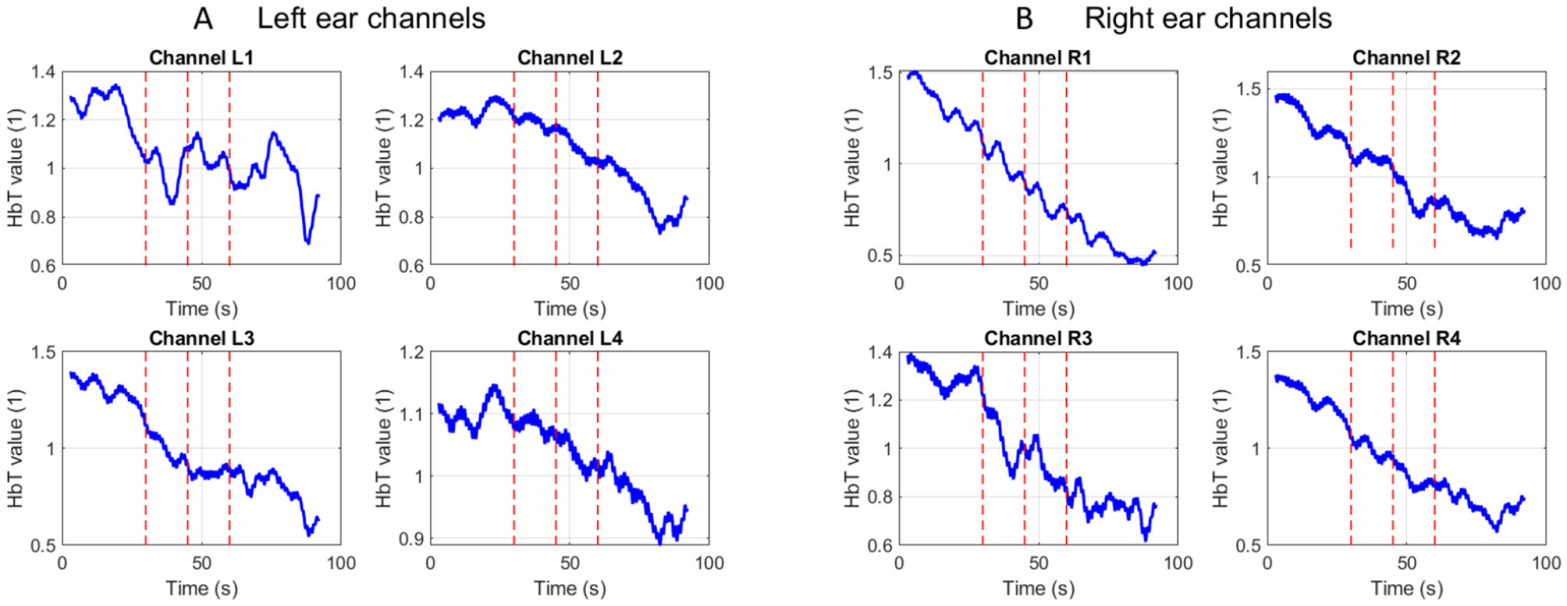
fNIRS “warble” —**(A)** left hemisphere response, **(B)** right hemisphere response.

Figure 5 illustrates representative Δ[HbT] time courses from channels overlying the right and left auditory areas. A consistent pattern observed following each “warble” stimulus presentation was an initial sharp decrease in the Δ[HbT] signal, followed by a subsequent increase in amplitude.

**Figure 6:**
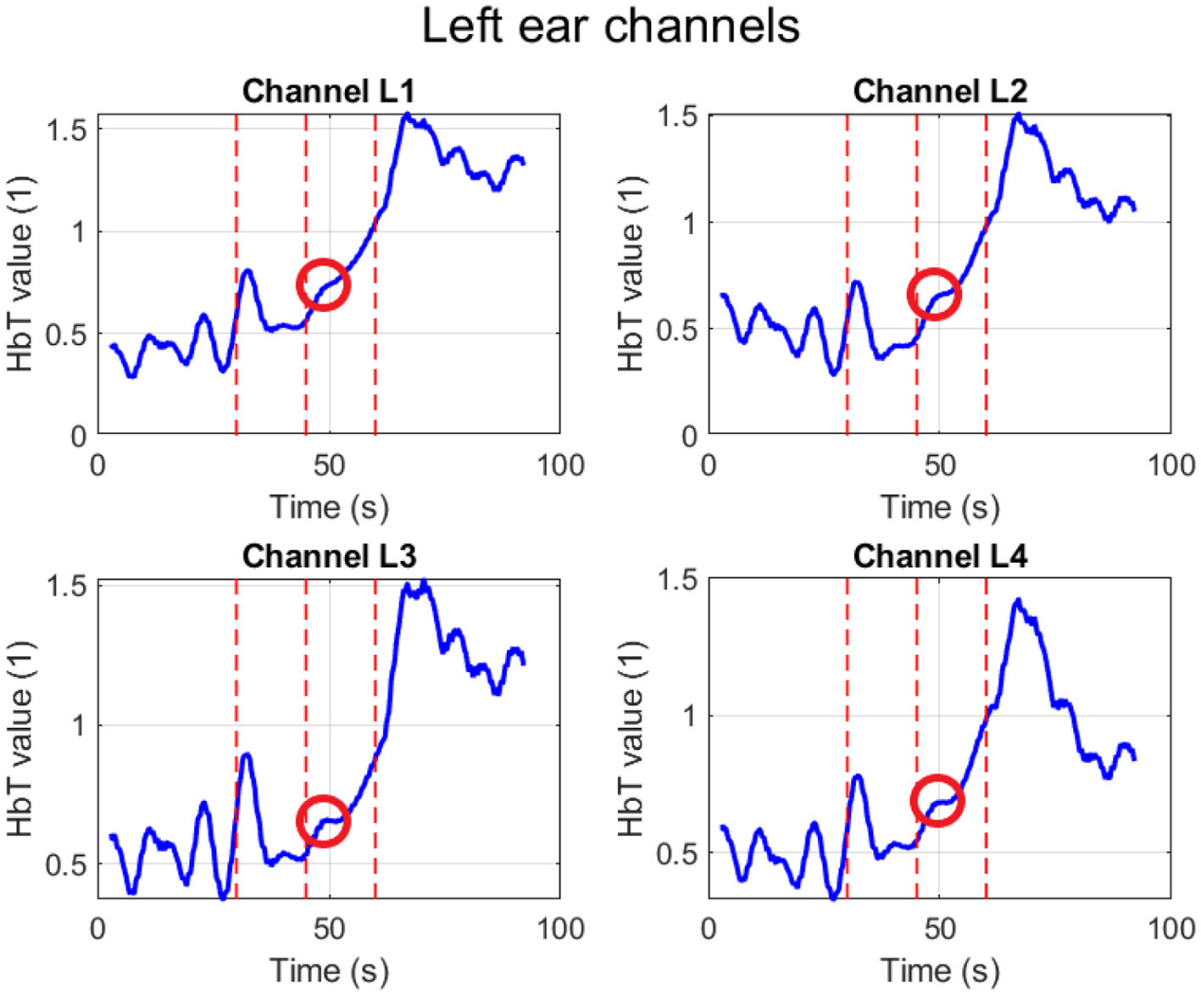
fNIRS “warble” — left hemisphere, breath-hold condition.

To investigate potential physiological confounds, additional measurements were conducted under a breath-hold condition. Figure 6 displays a representative recording from a left hemisphere channel during a breath-hold trial.

**Figure 7:**
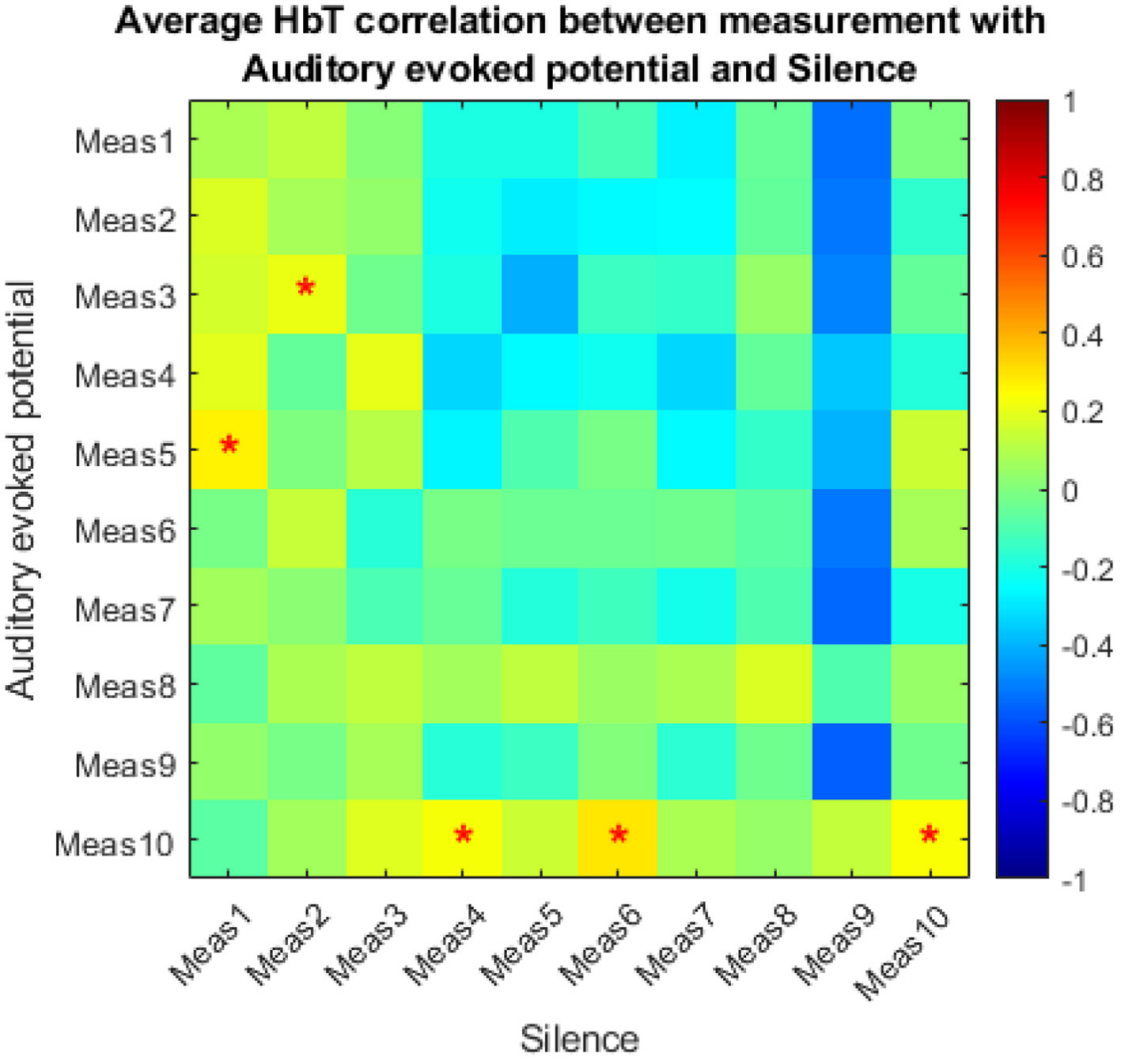
Average correlation values between Δ[HbT] signals during auditory stimulation periods and silence periods. Correlations above |r| > 0.25 are marked with asterisks.

The results of the correlation analysis are depicted in Figure 7. The matrix shows the average correlation values between the Δ[HbT] time courses during auditory stimulation and during silence. The majority of correlation coefficients ranged from slightly positive to negative, indicating a general dissimilarity between the signal time courses during stimulation and silence.

**Figure 8:**
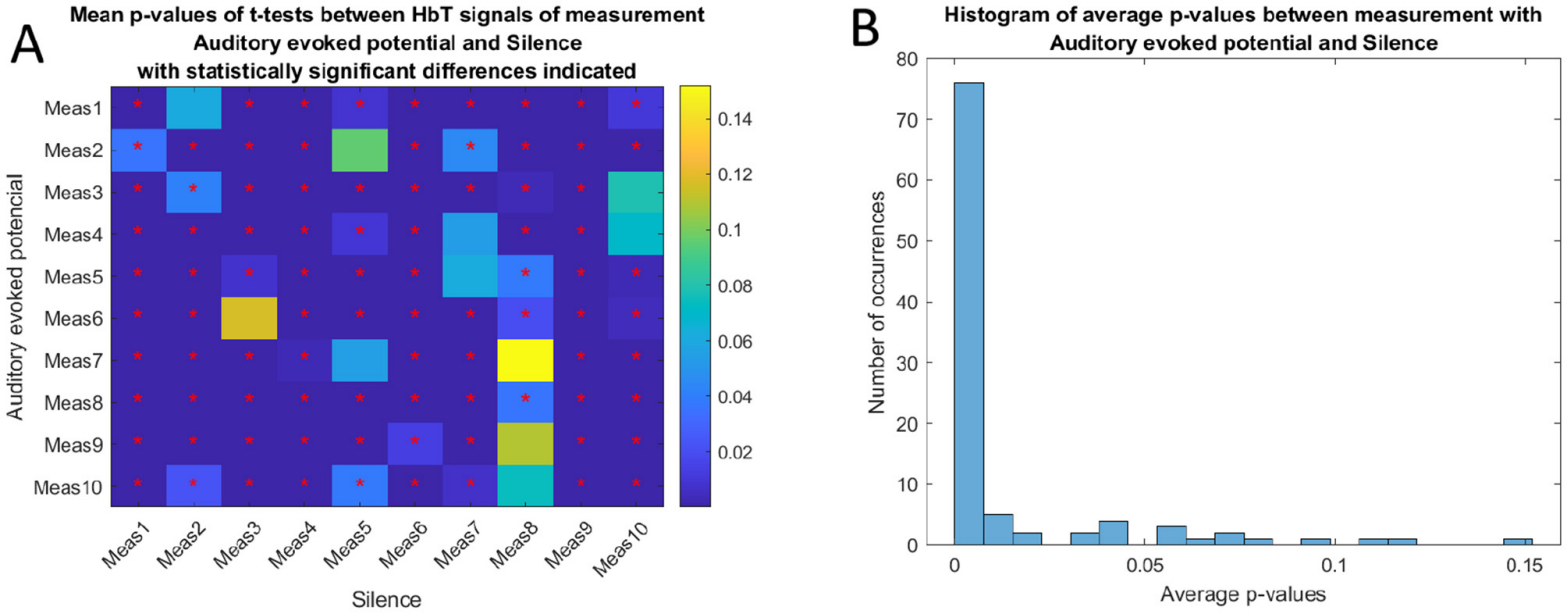
**(A)** Matrix of average *p*-values from t-tests between Δ[HbT] signals during auditory stimulation (AEP) periods and silence periods. Statistically significant comparisons (*p* < 0.05) are marked with red asterisks. **(B)** Histogram of *p*-values from *t*-tests between silence and auditory stimulation (AEP) periods.

To verify the statistical significance of these differences, *p*-values were calculated using *t*-tests. The significance threshold was set to α = 0.05. The resulting matrix of average *p*-values is shown in Figure 8A. Red asterisks indicate combinations with statistically significant differences (*p* < 0.05). The results indicate that the vast majority of comparisons show significant differences. The distribution of all calculated p-values is illustrated in the histogram in Figure 8B), where it is evident that the absolute majority of *p*-values fall below the 0.05 significance level.

**Figure 9:**
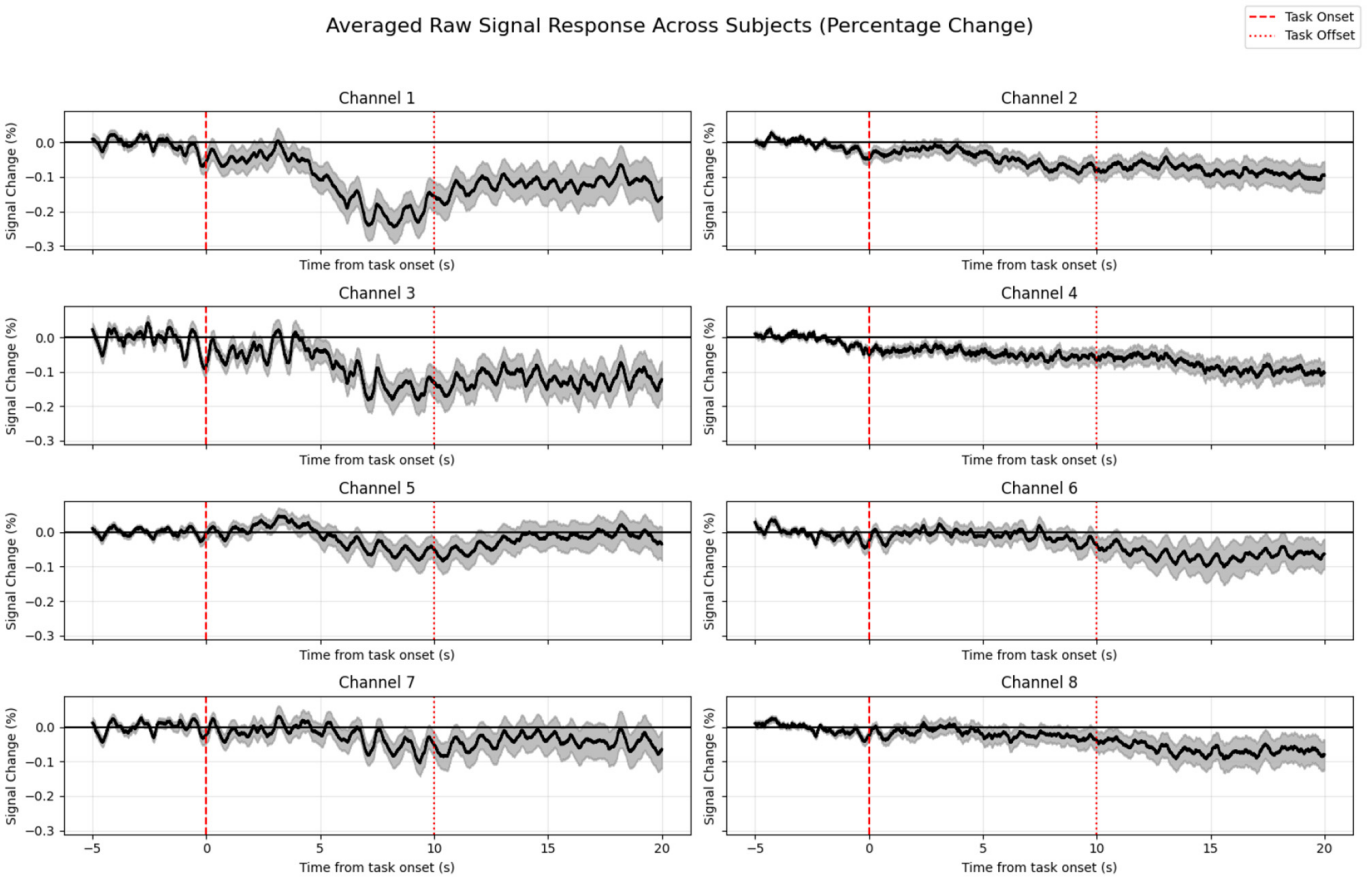
Averaged raw fNIRS signal response to the “warble” stimulus. The plot shows a consistent and sustained decrease in signal intensity (expressed as a percentage change from baseline) across all channels, clearly time-locked to the task onset (red dashed line at *t* = 0 s). The shaded area represents the standard error of the mean.

Visual inspection of the data, averaged across all participants and trials, showed a clear and unambiguous pattern. As illustrated in Figure 9, there is a distinct and sustained decrease in the raw signal intensity following the task onset. This decrease, which signifies increased light absorption in the tissue, reaches its minimum approximately 10–15 s after stimulus presentation before slowly returning toward baseline. This temporal profile is characteristic of a classic hemodynamic response.

**Figure 10:**
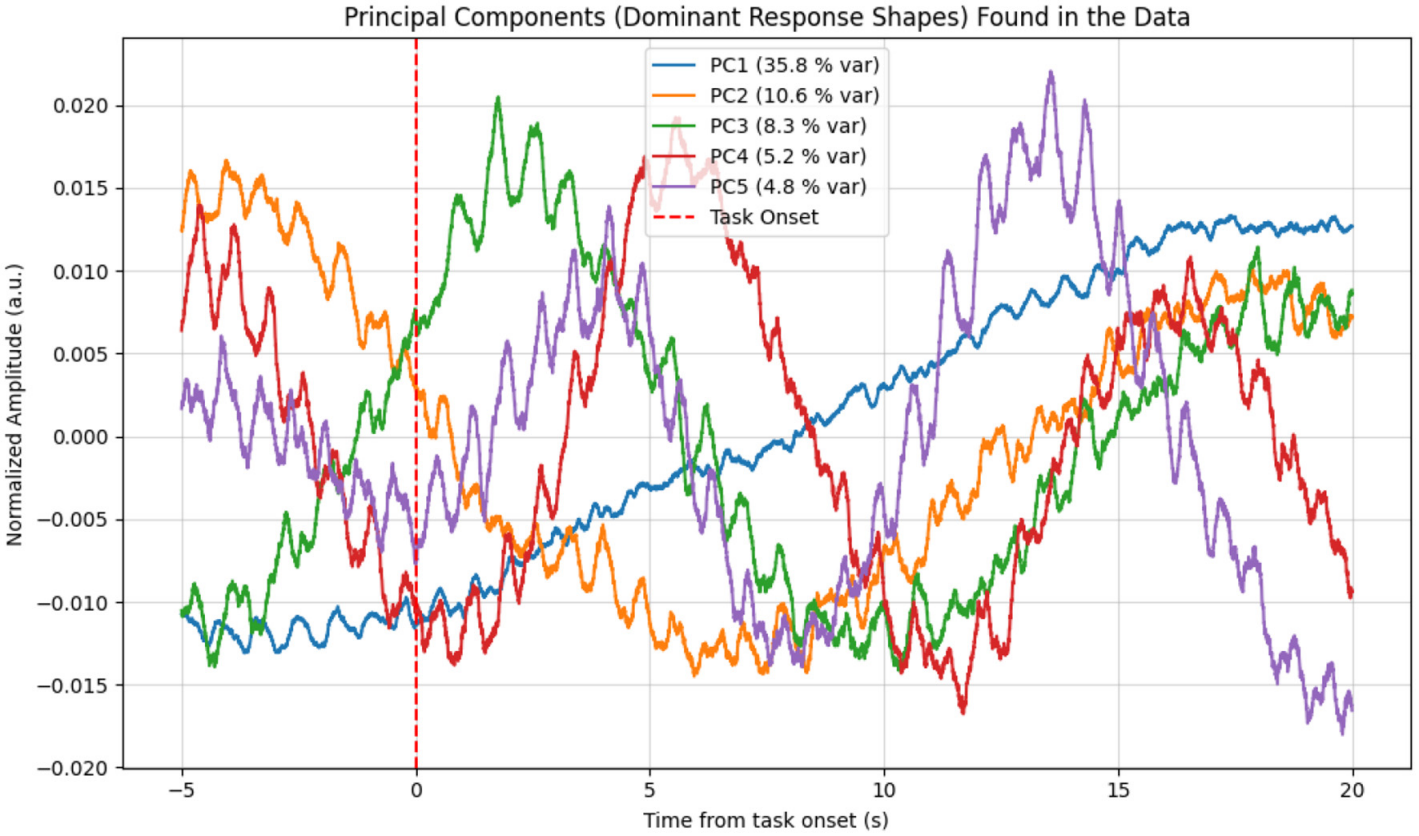
Principal components (PCs) extracted from the raw fNIRS data epochs. The first principal component (PC1, blue curve), which explains 35.8% of the data variance, shows a shape that almost perfectly matches the averaged hemodynamic response, with the characteristic decrease following the task onset. This provides data-driven confirmation of a consistent response pattern.

This characteristic response shape was further confirmed by Principal Component Analysis (PCA). As shown in Figure 10, the first principal component (PC1), which explained the largest portion of the variance in the data (35.8%), almost perfectly matched the time course of the averaged response. This data-driven finding indicates that the most dominant and frequently recurring pattern in the signal is indeed the response to the stimulus.

The statistical significance of this response was unequivocally validated by multiple methods. A General Linear Model (GLM) analysis confirmed that a model including the task regressor explained the data significantly better than a null model, yielding highly significant results across all channels (Table 2). This was further supported by a time-locked cross-correlation analysis which, as shown in Table 3, revealed a strong and statistically significant negative correlation. This is a key finding, as it explicitly confirms that the signal consistently and time-locked decreases in response to the stimulus.

**Table 2:** Results of the GLM analysis.

**Channel**	***t*-statistic**	***p*-value**	**Mean Beta**	***p*-value FDR corrected**	**Significant (TRUE/FALSE)**
Ch 1	6.835	0.000001	0.0142	0.000001	TRUE
Ch 3	7.076	0.000000	0.0227	0.000001	TRUE
Ch 6	7.157	0.000000	0.0173	0.000001	TRUE

All channels show is_significant = TRUE, indicating that the model including a task regressor explains the data significantly better than a model without it.

**Table 3:** Results of the cross-correlation analysis.

**Channel**	***t*-statistic**	***p*-value**	**Mean peak Correlation**	***p*-value FDR corrected**	**Significant (TRUE/FALSE)**
Ch 1	–24.887	0.0000	–1265.95	0.0000	TRUE
Ch 3	–25.592	0.0000	–1192.99	0.0000	TRUE
Ch 6	–23.431	0.0000	–1098.27	0.0000	TRUE

A strong and statistically significant negative correlation confirms that the signal consistently and time-lockedly decreases in response to the stimulus. Only a representative subset of results is shown.

**Figure 11:**
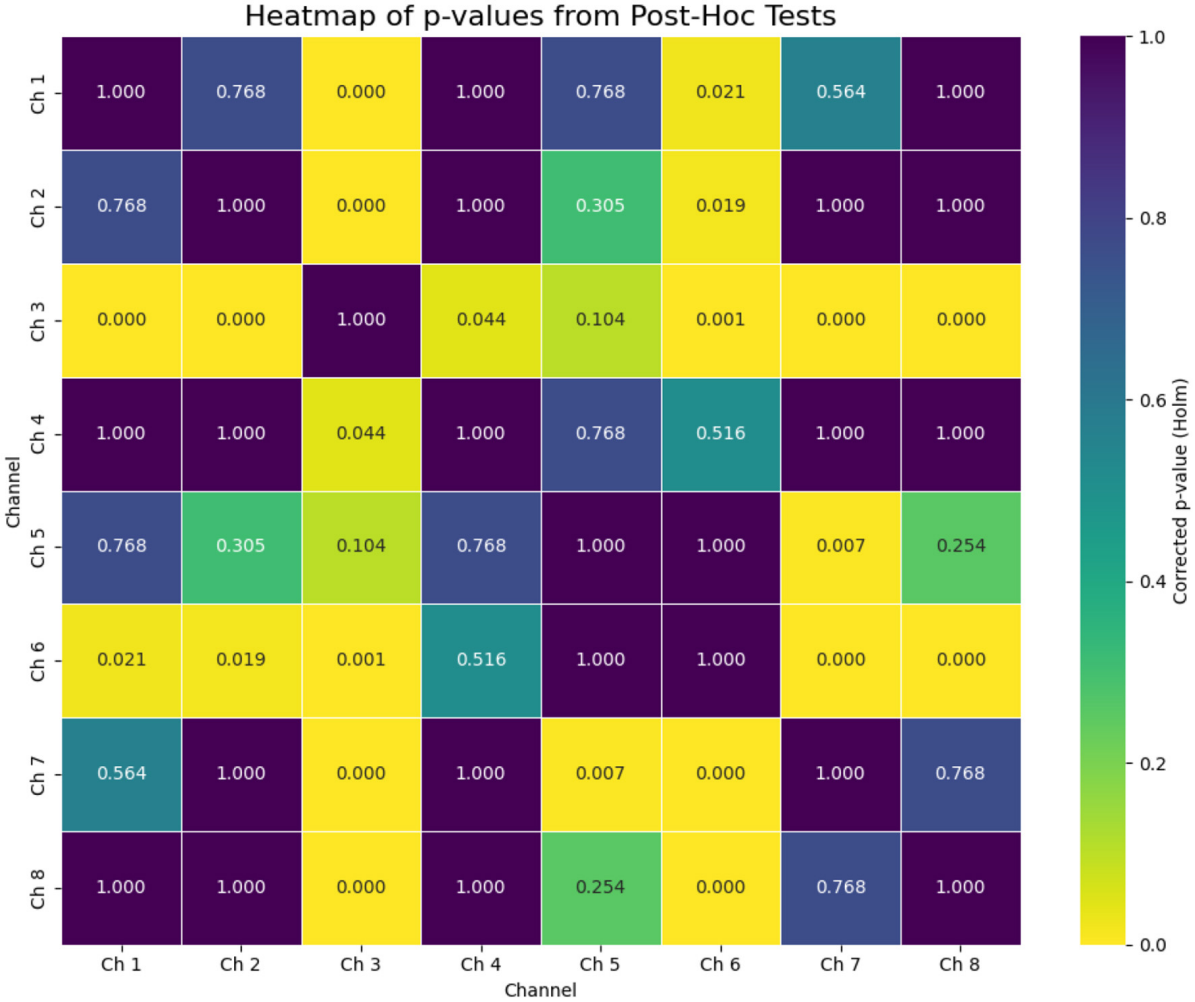
Heatmap of corrected *p*-values (Holm's method) from *post-hoc* pairwise tests comparing response strength (GLM beta-coefficients) between channels. Bright yellow indicates a statistically significant difference (*p* < 0.05) between a pair of channels, while dark purple indicates no significant difference. The distinct pattern confirms the ANOVA result of spatial specificity, arguing against a global artifact.

Crucially, the analysis of spatial specificity provided strong evidence for a neural origin of the signal. A repeated-measures ANOVA on the response amplitudes revealed a highly significant effect of channel [*F*(7, 154) = 7.74, *p* < 0.0001], with detailed results presented in Table 4. This indicates that the response strength was not uniform across the scalp but differed significantly between channels, a finding that argues strongly against a global systemic artifact (e.g., a change in blood pressure). *Post-hoc* tests, visualized in Figure 11, identified specific pairs of channels with significantly different response strengths, further supporting the hypothesis of localized brain activity, presumably originating from the auditory cortex. In summary, the ”battery of tests” provided converging evidence for a robust, repeatable, and spatially specific neural response to the warble stimulus, detectable with fNIRS.

**Table 4:** Results of the repeated-measures ANOVA for the “channel” factor.

**Effect**	***F*-value**	**Num DF**	**Den DF**	***p*-value (Pr > F)**
Channel	7.7357	7.0	154.0	< 0.0001

## 5 Discussion

The primary objective of this study was to conduct a direct comparison of two distinct neurotechnologies–the established electroencephalography (EEG) and the emerging functional near-infrared spectroscopy (fNIRS)–for measuring brain responses to auditory stimuli (Table 5). Our findings reveal a complementary relationship rather than a direct rivalry, with each modality demonstrating clear advantages within specific paradigms. The discussion of this comparison necessitates an evaluation from the perspectives of temporal resolution, methodological limitations, and practical applicability in clinical and research settings.

**Table 5:** Comparison of EEG and fNIRS methods for auditory evoked potential measurement.

**Criterion**	**EEG**	**fNIRS**
Temporal resolution	Very high (millisecond scale)	Low (second scale)
Spatial resolution	Moderate (depends on electrode montage and inverse solution)	Moderate (better localization in superficial cortex)
Artifact sensitivity	High (movement, muscle activity, EM interference)	Moderate (less sensitive to electrical noise; affected by respiration, HR, and movement)
System portability	Limited (larger devices, wired setup)	High (compact and wireless options)
Patient comfort	Lower (adhesive gel electrodes)	Higher (optical probes, usually gel-free)
Detection of rapid stimuli	Excellent (click ABR)	Insufficient (slow hemodynamic response)
Detection of slower stimuli	Good (cortical slow potentials)	Excellent (sustained hemodynamic changes)
Clinical standardization	High (widely accepted method)	Low (evolving and experimental)

EEG unequivocally affirmed its superiority in the time-precise detection of rapid, transient neural events, as demonstrated in our click-stimulus paradigm (Study 1). The identification of Jewett waves (I, III, and V) was successful, aligning with the vast body of literature on Auditory Brainstem Response (ABR) audiometry (1). It is important to note, however, that the observed latencies in our EEG recordings exhibited prolongations compared to normative values, as detailed in the results. Possible explanations include differences in stimulus calibration, reduced number of epochs (1,000 vs. the clinically recommended 3,000), and the relaxed participant state during testing, all of which may slightly prolong wave latencies. These factors should be considered when interpreting absolute latency values. In stark contrast, fNIRS proved unsuitable for resolving these millisecond-scale neuroelectric ABR events. This outcome is expected, given the fundamental biophysical mismatch: fNIRS measures a slow (on the order of seconds) metabolic and hemodynamic response, which cannot track the rapid (millisecond-scale) synchronized firing of brainstem neurons. The initial exploratory nature of Study 1, which yielded these largely negative findings for fNIRS in the ABR context, justified its smaller sample size.

Conversely, the true potential of fNIRS was unlocked in Study 2, which used a slower, more complex “warble” stimulus designed to elicit a sustained cortical response. For this paradigm, fNIRS unequivocally excelled, providing a robust and highly significant signal. The strength of this finding is bolstered by our multi-faceted analytical strategy; the convergence of evidence–from the clear pattern in the grand-averaged data, the dominant first principal component from PCA, and the high statistical significance in the GLM–provides compelling validation. Physiologically, the observed decrease in raw signal intensity corresponds to an increase in light absorption, which is the expected signature of a neurovascular response where increased neural activity in the auditory cortex leads to a local influx of oxygenated blood and a subsequent rise in total hemoglobin concentration.

Crucially, the repeated-measures ANOVA demonstrated that this hemodynamic response was spatially specific, with significantly different amplitudes across the optode channels. This result is of paramount importance, as it provides strong evidence against the signal being a mere global, systemic artifact (e.g., a uniform change in blood pressure, heart rate, or respiration) and instead supports its origin in localized neural activity within the auditory cortex. This spatial differentiation reinforces the validity of fNIRS as a genuine neuroimaging tool for auditory research.

### 5.1 Limitations of the study

Despite the promising results, this study has several limitations that must be acknowledged:

- **Sample Size Considerations**. The sample sizes of 20 participants for the click paradigm and 34 for the warble paradigm were selected based on both practical and statistical considerations. Given the expected medium-to-large effect sizes typically observed in auditory EEG and fNIRS studies (Cohen's *d*≈0.6–0.9), these group sizes provide adequate statistical power (approximately 0.8) to detect within-subject differences while keeping the total recording time feasible. The smaller cohort in the click paradigm was further justified by its exploratory nature and shorter recording duration, whereas the larger sample for the warble paradigm was intended to ensure robust estimation of hemodynamic effects.
- **Reduced Epoch Count for ABR:** The ABR protocol (Study 1) utilized 1,000 stimulus repetitions for averaging. While this was a pragmatic choice to simulate time-constrained pediatric assessments, it is substantially lower than the 2,000 or more epochs often recommended in clinical practice to achieve an optimal signal-to-noise ratio (SNR) for clear wave identification (33). This may have contributed to the observed variability and increased latencies in our EEG recordings.
- **Homogeneous Participant Cohort:** This study was conducted exclusively on a cohort of healthy, normal-hearing adult volunteers. The findings, particularly the robust fNIRS response to the “warble” stimulus, have not yet been validated in a clinical population with hearing impairments, cochlear implant users, or individuals with neurological or developmental disorders. Therefore, the clinical generalizability of our results remains to be established.
- **Basic Artifact Handling:** Our fNIRS data processing pipeline relied on standard filtering and averaging, with spatial specificity confirmed via ANOVA. However, it did not include more advanced artifact correction methods, such as short-channel regression to remove scalp hemodynamics (34), or component-based filtering (e.g., ICA) to separate neural signals from systemic physiological noise like respiration and cardiac pulsations. The absence of these techniques means that subtle contamination from non-neural sources cannot be fully ruled out. Future implementations of our analysis pipeline will include short-channel regression and component-based filtering to further improve separation of neural and systemic signals.
- **Limited Stimulus Variety:** The study was constrained to two specific stimulus types: a broadband click and a custom ”warble” sound. While effective for demonstrating the core differences between EEG and fNIRS capabilities, this does not explore the full range of auditory processing. The response to other clinically relevant stimuli, such as speech-in-noise or pure tones, was not assessed.

### 5.2 Future directions

The findings of this study open several exciting avenues for future research that can build upon our work and address its limitations. Key directions include:

- **Clinical Validation Studies:** The most critical next step is to test the fNIRS “warble” paradigm in clinical populations. Studies involving individuals with varying degrees and types of hearing loss, patients with cochlear implants, and children with suspected auditory processing disorders are needed to establish its diagnostic utility, sensitivity, and potential for applications like objective hearing aid fitting or tracking auditory rehabilitation.
- **Advanced Signal Processing and Machine Learning:** Future work should focus on implementing sophisticated preprocessing pipelines. This includes integrating algorithms for movement artifact removal and employing data-driven techniques like Independent Component Analysis (ICA) to better isolate neural responses. Furthermore, the application of machine learning models could enable the automatic classification of responses, prediction of hearing thresholds from fNIRS data, and differentiation between pathologies, leading to more objective and automated diagnostic workflows.
- **Stimulus Optimization for fNIRS:** A systematic investigation into a wider range of auditory stimuli is warranted. Exploring responses to speech syllables, amplitude-modulated tones, and stimuli presented in background noise (35) could help characterize the specific acoustic features that elicit the most robust and informative cortical hemodynamic responses, allowing for the design of targeted diagnostic protocols.
- **True Multimodal Integration:** Moving beyond simple parallel recording, future studies should aim for true data fusion, where EEG and fNIRS signals are combined within a unified analytical framework. Such an approach could use the high temporal resolution of EEG to inform the interpretation of the fNIRS signal, providing deeper insights into the dynamics of neurovascular coupling in both healthy and disordered auditory systems (36).

In summary, while EEG remains the indispensable gold-standard for rapid brainstem diagnostics, fNIRS emerges as a powerful complementary tool for assessing cortical auditory processing. Its practical advantages–including portability, greater patient comfort, and robustness to certain artifacts–make it an exceptionally promising modality for expanding the reach of objective audiological assessment to new populations and environments, particularly in pediatric cohorts or individuals where traditional EEG is challenging.

## 6 Conclusion

This study systematically compared EEG and fNIRS for measuring brain responses to auditory stimuli, revealing a clear division of utility rather than a direct rivalry. Our findings unequivocally confirm that EEG, with its superior temporal resolution, remains the indispensable gold standard for rapid auditory brainstem diagnostics. It successfully captured the millisecond-scale Jewett waves in response to click stimuli, a task for which fNIRS proved fundamentally unsuitable due to the slow nature of the hemodynamic response.

Conversely, this research demonstrates the significant potential of fNIRS in a different domain. When presented with slower, more complex “warble” stimuli, fNIRS excelled, detecting a robust, repeatable, and spatially specific cortical hemodynamic response in all participants. Rigorous statistical analysis validated this response as being of neural origin, effectively ruling out systemic physiological artifacts and establishing the method's validity for this type of assessment.

The implications of these findings are twofold. First, they delineate clear operational boundaries for each technology: EEG is the tool of choice for high-fidelity temporal analysis of brainstem pathways, while fNIRS is a powerful instrument for assessing sustained cortical processing. Second, and perhaps more importantly, the demonstrated strengths of fNIRS–its non-invasiveness, patient comfort, and portability–position it as a highly valuable complementary tool. It holds particular promise for clinical scenarios where traditional EEG is constrained, such as in objective audiological assessments for pediatric populations, cochlear implant users, or in mobile, out-of-clinic settings.

In conclusion, this work establishes that fNIRS is not a replacement for EEG in audiology but rather a powerful adjunct. By leveraging the unique strengths of each modality, clinicians and researchers can achieve a more comprehensive understanding of the entire auditory pathway, from brainstem to cortex. The successful application of fNIRS in our study paves the way for its integration into clinical practice, where it can enhance diagnostic capabilities, improve the patient experience, and foster new research into the cortical dynamics of hearing.

## Data Availability

The original contributions presented in the study are included in the article/supplementary material, further inquiries can be directed to the corresponding author/s.

## References

[1] Radeloff A Cebulla M Shehata-Dieler W. Akustisch evozierte Potenziale: Grundlagen und klinische Anwendung. Laryngo-Rhino-Otologie. (2014) 93:625–37. doi: 10.1055/s-0034-1385868 25152975

[2] Jerger J Lew HL. Principles and clinical applications of auditory evoked potentials in the geriatric population. Phys Med Rehabil Clin N Am. (2004) 15:235–50. doi: 10.1016/S1047-9651(03)00099-8 15029907

[3] Cañete SO. Potenciales evocados auditivos de corteza: Complejo P1-N1-P2 y sus aplicaciones clínicas. Rev Otorrinolaringol Cirugía Cabeza Cuello. (2014) 74:266–74. doi: 10.4067/S0718-48162014000300012

[4] Gelaney AE Mostafa EM Abdelmawgoud SM Ahmed Ibrahim MA. Auditory brainstem response in children with attention deficit hyperactive disorder. Egypt J Ear Nose Throat Allied Sci. (2024) 25:1–6. doi: 10.21608/ejentas.2024.293218.1757

[5] Bálint A Wimmer W Caversaccio M Rummel C Weder S. Brain activation patterns in normal hearing adults: an fNIRS Study using an adapted clinical speech comprehension task. Hear Res. (2025) 455:109155. doi: 10.1016/j.heares.2024.109155 39637600

[6] Lawrence RJ Wiggins IM Anderson CA Davies-Thompson J Hartley DEH. Cortical correlates of speech intelligibility measured using functional near-infrared spectroscopy (fNIRS). Hear Res. (2018) 370:53–64. doi: 10.1016/j.heares.2018.09.005 30292959

[7] Steinmetzger K Megbel E Shen Z Andermann M Rupp A. Cortical activity evoked by voice pitch changes: a combined fNIRS and EEG study. Hear Res. (2022) 420:108483. doi: 10.1016/j.heares.2022.108483 35305854

[8] Zhang W Yu Z Ruan Q. Presbycusis-Related Tinnitus and Cognitive Impairment: Gender Differences and Common Mechanisms. London: IntechOpen (2020). doi: 10.5772/intechopen.90956

[9] [Dataset] Wichita State University. Facilities & Lab Equipments (2023). Available online at: https://www.wichita.edu/research/bme/Facilities___Lab_Equipments.php (Accessed August 10, 2023).

[10] [Dataset] GTec Medical Engineering GmbH. fNIRS Sensor (2023). Available online at: https://www.gtec.at/product/fnirs-sensor/ (Accessed August 10, 2023).

[11] Michel CM Koenig T. EEG microstates as a tool for studying the temporal dynamics of whole-brain neuronal networks: a review. Neuroimage. (2018) 180:577–93. doi: 10.1016/j.neuroimage.2017.11.062 29196270

[12] [Dataset] Wikipedia Contributors. File:21 Electrodes of International 10-20 System for EEG.svg (2023). Available online at: https://commons.wikimedia.org/wiki/File:21_electrodes_of_International_10-20_system_for_EEG.svg?uselang=en#Licensing (Accessed May 12, 2023).

[13] Lefler SM Kaf WA Ferraro JA. Comparing simultaneous electrocochleography and auditory brainstem response measurements using three different extratympanic electrodes. J Am Acad Audiol. (2021) 32:339–46. doi: 10.1055/s-0041-1727273 34082461

[14] Xia W Song D Fu H Lei T Wang K Zeng Y. Comparison of subdermal needle and surface adhesive electrodes for intraoperative neuromonitoring during spine surgeries. J Orthopaed Surg Res. (2025) 20:490. doi: 10.1186/s13018-025-05907-9 40394618 PMC12090551

[15] [Dataset] Sennheiser. HD 280 PRO (506845) (2023). Available online at: https://www.sennheiser.com/en-at/catalog/products/headphones/hd-280-pro/hd-280-pro-506845 (Accessed August 10, 2023).

[16] [Dataset] Brüel & Kjær. Type 4157 Artificial Ear Simulator (2023). Available online at: https://www.bksv.com/de/transducers/simulators/ear-mouth-simulators/4157 (Accessed August 10, 2023).

[17] The The MathWorks Inc. MATLAB. Natick, MA (2023). Version R2023a. Available online at: https://www.mathworks.com/products/matlab.html (Accessed August 10, 2023).

[18] Eggermont JJ. Neuroaudiology. Cambridge University Press (2012).

[19] Vivek KV Chhina AS. A comparative analysis of otoacoustic emission and automated auditory brainstem response for newborn hearing screening. Indian J Pediatr. (2024) 91:1209. doi: 10.1007/s12098-024-05156-4 38730120

[20] Manta O Sarafidis M Vasileiou N Schlee W Consoulas C Kikidis D . Development and evaluation of automated tools for auditory-brainstem and middle-auditory evoked potentials waves detection and annotation. Brain Sci. (2022) 12:1675. doi: 10.3390/brainsci12121675 36552135 PMC9775187

[21] Lentz JJ Walker MA Short CE Skinner KG. Audiometric testing with pulsed, steady, and warbletones in listeners with tinnitus and hearing loss. Am J Audiol. (2017) 26:328–37. doi: 10.1044/2017_aja-17-0009 28892822 PMC5831060

[22] Luke R Larson E Shader MJ Innes-Brown H Van Yper L Lee AKC . Analysis methods for measuring passive auditory fnirs responses generated by a block-design paradigm. Neurophotonics. (2021) 8:025008. doi: 10.1117/1.nph.8.2.025008 34036117 PMC8140612

[23] Bonilauri A Sangiuliano Intra F Baselli G Baglio F. Assessment of fnirs signal processing pipelines: towards clinical applications. Appl Sci. (2022) 12:316. doi: 10.3390/app12010316

[24] Ong JH Chia KS. Principal components-artificial neural network in functional near-infrared spectroscopy (fnirs) for brain control interface. In: 2021 International Conference on Innovation and Intelligence for Informatics, Computing, and Technologies (3ICT) (Zallaq: IEEE) (2021). p. 714–9. doi: 10.1109/3ICT53449.2021.9581861

[25] von Lühmann A Li X Müller KR Boas DA Yücel MA. Improved physiological noise regression in fnirs: a multimodal extension of the general linear model using temporally embedded canonical correlation analysis. NeuroImage. (2020) 208:116472. doi: 10.1016/j.neuroimage.2019.116472 31870944 PMC7703677

[26] Izzetoglu M Holtzer R. Evaluation of neural, systemic and extracerebral activations during active walking tasks in older adults using fnirs. IEEE Trans Neural Syst Rehabil Eng. (2025) 33:807–17. doi: 10.1109/TNSRE.2025.3540673 40031581 PMC12054330

[27] Walia P Fu Y Norfleet J Schwaitzberg SD Intes X De S . Error related fnirs-eeg microstate analysis during a complex surgical motor task. In: 2022 44th Annual International Conference of the IEEE Engineering in Medicine & Biology Society (EMBC) (Piscatway, NJ: IEEE) (2022). p. 941–4. doi: 10.1109/EMBC48229.2022.9871175 36083946

[28] Lu J Zhang X Wang Y Cheng Y Shu Z Wang J . An fnirs-based dynamic functional connectivity analysis method to signify functional neurodegeneration of Parkinson's disease. IEEE Trans Neural Syst Rehabil Eng. (2023) 31:1199–207. doi: 10.1109/TNSRE.2023.3242263 37022412

[29] Dang JC Hsu NM. Hearing Loss Screening Guidelines. Treasure Island, FL: StatPearls Publishing (2023). Available online at: https://www.ncbi.nlm.nih.gov/books/NBK597360/ (Accessed November 5, 2023). 37983360

[30] Burkard RF Eggermont JJ Don M. Auditory Evoked Potentials: Basic Principles and Clinical Application. Lippincott Williams & Wilkins (2007).

[31] Oh SH Lee J. A systematic review of audiology terminology. J Audiol Otol. (2016) 20:109–13. doi: 10.7874/jao.2016.20.2.109 27626085 PMC5020571

[32] Bohórquez J Özdamar O. Signal to noise ratio analysis of maximum length sequence deconvolution of overlapping evoked potentials. J Acoust Soc Am. (2006) 119:2881–8. doi: 10.1121/1.2191609 16708946

[33] Suzuki J Kodera K Kaga K. Auditory evoked brainstem response assessment in otolaryngology. Ann N Y Acad Sci. (1982) 388:487–500. doi: 10.1111/j.1749-6632.1982.tb50811.x 6953884

[34] Cooper RJ Selb J Gagnon L Phillip D Schytz HW Iversen HK . A systematic comparison of motion artifact correction techniques for functional near-infrared spectroscopy. Front Neurosci. (2012) 6:147. doi: 10.3389/fnins.2012.00147 23087603 PMC3468891

[35] Lawrence RJ Wiggins IM Hodgson JC Hartley DE. Evaluating cortical responses to speech in children: a functional near-infrared spectroscopy (fnirs) study. Hear Res. (2021) 401:108155. doi: 10.1016/j.heares.2020.108155 33360183 PMC7937787

[36] Li R Yang D Fang F Hong KS Reiss AL Zhang Y. Concurrent fnirs and eeg for brain function investigation: a systematic, methodology-focused review. Sensors. (2022) 22:5865. doi: 10.3390/s22155865 35957421 PMC9371171

